# Factors Influencing Public Donation Intention during Major Public Health Emergencies and Their Interactions: Evidence from China

**DOI:** 10.3390/bs14100927

**Published:** 2024-10-10

**Authors:** Minghua Zhao, Beihai Tian

**Affiliations:** College of Humanities and Social Sciences, Huazhong Agricultural University, Wuhan 430070, China; zhaomh@webmail.hzau.edu.cn

**Keywords:** major public health emergency, public donation intention, hardship level caused by the pandemic, degree of risk perception, community material support, evaluation of the pandemic response

## Abstract

The COVID-19 pandemic is a major public health emergency that has caused significant global devastation. However, it has also fostered unprecedented worldwide solidarity. During this crisis, we have witnessed large-scale donations and assistance both domestically and internationally. In the face of such extensive public engagement, understanding the driving factors behind public donations is crucial in responding to future global shocks like the COVID-19 pandemic. This study proposes an analytical framework and examines the factors influencing public donation intention during major public health emergencies and their interactions. Based on the online and telephone survey data of 11,682 responses collected in China during the COVID-19 pandemic in 2020, this study employs multiple logistic regression and moderation effect models to analyze these influencing factors and their interactions on public donation intention. The findings reveal a remarkably high level of public engagement, with 79% of respondents expressing donation intention. Further analysis indicates that the hardship level caused by the pandemic, degree of risk perception, community material support, and evaluation of the pandemic response all have a significant and positive impact on public donation intention. Moreover, the evaluation of the pandemic response and community material support significantly and positively moderate the impact of the hardship level caused by the pandemic and degree of risk perception on public donation intention, respectively. This study provides valuable guidance for governments and organizations worldwide. It is helpful for enriching crisis management theory and improving crisis response mechanisms.

## 1. Introduction

In early 2020, the COVID-19 pandemic emerged as a major global public health emergency, and its rapid spread and severe impact triggered widespread concern and urgent responses across the globe. Facing the ferocious pandemic, many countries implemented various measures for prevention and control. We have seen unprecedented global solidarity in this crisis, including large-scale donations and assistance both domestically and across borders [1]. Understanding the driving factors behind public donations is crucial in responding to global shocks like the COVID-19 pandemic. China, as one of the first countries affected by the outbreak, presents a valuable opportunity for empirical investigation into public donation intention during a significant public health crisis. The 2020 China Charity Donation Report revealed that China received a total of CNY 225.313 billion in charitable donations from both domestic and international sources, with enterprises and individuals contributing CNY 121.811 billion and CNY 52.415 billion, respectively. Health and wellness were the main donation areas in 2020, receiving a total of CNY 71.036 billion in donations, a 160.94% increase from the previous year, accounting for 34.05% of the total. Donations for pandemic control accounted for the largest share, with a total of CNY 39.627 billion in funds and 1.09 billion items received in the first half of 2020 alone (Data Source: China Charity Alliance 2020 Annual Report on Charitable Donations in China (Abridged Version). Available at http://www.charityalliance.org.cn/news/14364.jhtml (accessed on 20 October 2022)). During sudden disasters, public donation behavior becomes a key supplement to pandemic control. In crisis governance, philanthropic donations can not only quickly gather funds and materials, reducing government crisis management costs and improving efficiency, but also rapidly enhance social cohesion [2]. Broadly mobilizing the public for charitable donations during a crisis can fully activate social resources, compensating for the shortage of public resources and providing significant support for the government in responding to regional and national emergencies [3]. Disastrous events often trigger public donation enthusiasm and significantly increase individual donation levels [4]. However, the mechanisms at play are not yet clear and require further research. Donation intention reflects an individual attitude towards and potential commitment to donate, while donation behavior is the result of this intention being converted into action. Therefore, donation intention is often seen as a precursor or predictive factor for donation behavior. Theories of reasoned action [5] and planned behavior [6] suggest that intention is a psychological tendency or internal drive that prompts individuals towards a specific goal, making it the most direct predictor of behavior. Numerous analyses have found that intention offers superior prediction of behavior in correlational tests compared to other cognitions including (explicit and implicit) attitudes, norms, self-efficacy, and perceptions of risk and severity as well as personality factors [7]. Exploring and identifying the public donation intention during major public health emergencies is crucial for guiding resource allocation, improving donation efficiency, optimizing policy formulation, and enhancing social unity. This study delves into the mechanisms influencing public donation intention from the perspectives of individual circumstances, community support, and government actions, revealing the internal logic behind public donation intention in the face of public health crises. Theoretically, this research helps to discern the influence mechanism of public donation intention during crises from individual and organizational perspectives. Practically, it can guide the government in effectively mobilizing the public, optimizing crisis response and resource allocation, and promoting social unity and public participation, thereby enhancing the government’s ability to manage public health crises. The findings not only provide new theoretical and empirical support for understanding public donation behavior during major public health emergencies but also offer valuable guidance for the government and communities in mobilizing and utilizing public resources more effectively in future public health emergencies.

## 2. Literature Review

Donation intention has been extensively studied across various disciplines such as sociology, economics, psychology, and management. This study reviews the existing research on donation intention from individual, institutional, and cultural perspectives based on a comprehensive analysis of the literature.

From the individual perspective, studies on donation intention focus on personal characteristics, emotional orientation, and trust. In terms of individual characteristics, factors such as the donor’s gender, marital status, education level, income, and religious beliefs have significant explanatory power for their donation intention and behavior [8]. It is observed that women are more likely to make charitable donations than men, married individuals more so than unmarried ones [9]; higher education levels increase the likelihood of donating; higher individual income levels correspond to stronger donation intention [10]; and stronger religious beliefs contribute more significantly to charitable donations [11]. Additionally, research indicates that an individual’s sense of power influences donation intention through situational prevention and promotion focus [12]. Emotional orientation includes sympathy, empathy, and awe. Studies on sympathy affirm that it is a universal emotion, leading to the conclusion that the motivation for charitable donations originates from sympathy. Adam Smith in *The Theory of Moral Sentiments* noted, “We often feel sad when we see others in sorrow, which is an obvious and undeniable fact” [13]. Many scholars believe that humaneness, sympathy, and compassion are the roots of the spirit and behavior of public charity, with sympathy for vulnerable groups being an important motivation for philanthropic actions [14]. People with more sympathy tend to donate and volunteer more [15]. External information stimuli, such as the portrayal of the recipient’s tragic circumstances in donation advertisements, can evoke the donor’s sympathy, thereby increasing their donation behavior [16]. Moreover, the severity of collective threats (such as environmental pollution and pandemic outbreaks) positively affects people’s donation intention. Specifically, when a collective threat becomes more severe, people under threat tend to pay more attention to and feel empathy for others experiencing the same threat, thus increasing their donation intention [17]. The research on empathy orientation debates the type of helping motivation it triggers, whether it is for alleviating one’s own negative state (egoistic) or for relieving the victim’s suffering (altruistic). Batson and colleagues, after analyzing a large body of evidence, proposed that pure (i.e., unselfish) altruism occurs in the context of empathy. In contrast, R.B. Cialdini et al. demonstrated through experimental methods that the helping motivation arising from empathy is aimed at reducing one’s own distress (egoism) [18]. Batson responded to R.B. Cialdini’s conclusion with experimental studies, asserting that altruistic behavior is rooted in empathy for others, supporting the empathy–altruism hypothesis [19]. Both sympathy and empathy are human emotions. Comprehensive studies based on the help-decision model and affective adaptation theory have validated the dynamic psychological mechanism of “stimulus → emotion → motivation → intention”, indicating that whether motivated by egoism or altruism, individual charitable donations are results induced by emotions [20], and positive and negative emotions can both drive donating [21]. Research on awe suggests that it is a positive emotion that can motivate individuals towards spiritual pursuits and self-transcendence, shifting attention from oneself to others and the collective good [22]. Sporadic feelings of awe can evoke an individual motivational state or lead to inferences about the recipient’s lack of self-rescue ability, thereby enhancing the donor’s donation intention [23]. Trust serves as a fundamental determinant in shaping individuals’ propensity to contribute to charitable organizations. Empirical evidence consistently demonstrates that trust in philanthropic entities stands as a robust predictor of donation intentions and behaviors [24]. The significance of trust is further amplified in the digital era, where online donations constitutes a sizable and rapidly growing segment of the market for charitable giving [25]. In online donation platforms, both quality aspects (such as system quality and information quality) and institutional mechanism aspects (such as perceived platform rules and perceived monitoring) influence trust and distrust [26]. Trust in online donation platforms can positively predict donation intention [27].

From the institutional perspective, studies on charitable donation intention focus on promoting fairness and enhancing efficiency. During normal times, the charitable donation intention from an institutional perspective revolves around the promotion of fairness. Institutions that ensure fairness themselves constitute external factors affecting individual charitable donations. Donors motivated by the maintenance of social justice pay more attention to the fairness of distribution and take action to reduce injustice [28]. Tax policy is an important variable affecting charitable donations, and it is generally believed that tax incentives can effectively stimulate more charitable donations [29]. Estate tax is a significant motivation for many wealthy individuals to make charitable donations before their death, and many scholars abroad have analyzed the impact of tax policy on charitable donation motivation from the perspective of estate tax. The imposition of estate tax encourages the wealthy to donate, and regions with tax incentives stimulate more enthusiasm for donations among the wealthy compared to regions without tax incentives [30]. For ordinary citizens, engaging in charitable donations can result in direct economic benefits from tax deductions, which do not vary with the motivation or quality of the citizens but differ due to the tax policies of different countries and regions. Formulating reasonable pre-donation tax deduction policies is conducive to promoting charitable donations among citizens [31]. In terms of other measures to promote donations, research indicates that the use of the word ‘donation’ generated higher revenue than the use of ‘contribution’ [32]. Furthermore, providing matching funds for all donations can substantially increase overall donation rates [33]. The matching donation mechanism can also encourage more low-income individuals to participate in charitable donation [34]. In extraordinary times, institutional-based research on charitable donations focuses on institutional optimization to enhance donation efficiency. Charitable donations during extraordinary times exhibit different characteristics and forms compared to previous donations. This requires the government to improve the standardization of charitable donations and play an organizational coordination role to maximally mobilize enthusiasm for donations from all walks of life, ensuring the rational distribution of donated funds and goods. While charity organizations positively impact our societies, charity misconduct on public health issues has significantly reduced individuals’ intention to offer help via both the charity involved with the misconduct and any charity they prefer [35].

From the cultural perspective, studies on donation intention revolve around morality and responsibility. Social culture acts as an intrinsic motivation for public charitable donations [36], with religious culture having a significant impact on charitable donations in Western countries, while traditional culture has a more pronounced influence on charitable donations in China [37]. Chinese traditional culture has always emphasized concepts such as “joy in benevolence”, “aiding those in peril and difficulty”, and “repaying even a drop of kindness with a spring of benevolence”, which are the cultural roots of the Chinese people’s benevolent actions [38]. Charitable thoughts from Confucian “benevolence”, Mohist “universal love”, Taoist “accumulation of virtue”, and Buddhist “compassion” have jointly shaped the virtue of “joy in benevolence” within the Chinese nation, becoming an important ideological source for the rise and development of China’s charitable cause. The root of philanthropic behavior is the moral responsibility that one should bear in social life, with individual contributions to public welfare benefiting future generations [39]. The sense of social responsibility among citizens is the foundation for promoting mutual aid in society. In times of crisis, many businesses, families, and individuals donate out of a strong sense of social responsibility [40], and those who take action against COVID-19 and poverty are more willing to donate [41]. Even if some individuals have limited economic resources, they may still suppress their own needs to fulfill their social responsibilities, even willing to make sacrifices [42]. In Western countries, religious beliefs endow believers with stronger altruistic and social responsibility values, prompting them to be more generous towards charitable secular causes [43]. Many believers start with the interests of the church for their donations and volunteer services, and on this basis, engage in other charitable activities [44]. Eleanor Brown classifies American charitable donations into religious giving, secular giving, and volunteer services, finding through empirical research that devout believers donate more [9].

The rich research on donation intention primarily focuses on the public’s regular donation behavior during normal times. However, due to the suddenness, specificity, and rarity of disasters during crisis periods, the existing literature lacks a systematic study on the public donation intention during major public health emergencies in China. This gap is partly due to the difficulty in obtaining research subjects and materials for donation intention studies during extraordinary times and partly because, during crises, the focus is mostly on improving the tax system related to donations and the standardized operation of charitable organizations, leading to insufficient attention to public donation intention. Based on this, this paper focuses on the government’s pandemic prevention measures and the public risk experience during the COVID-19 pandemic from the perspectives of pandemic response evaluation, community material support, degree of risk perception, and hardship level caused by the pandemic, to analyze the influencing factors of public donation intention and thereby reveal the deep-seated factors of public donation intention during the major public health emergency, providing countermeasure suggestions to enhance the government’s emergency response capabilities in future public emergencies.

## 3. Theoretical Analysis and Research Hypotheses

### 3.1. Theoretical Analysis

**Figure 1:**
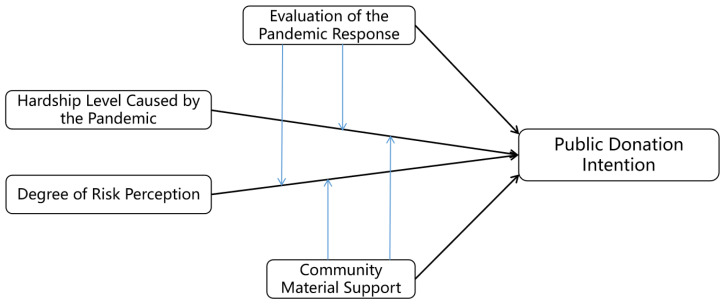
Analytical framework.

Public donation intention is influenced by a complex mechanism involving multiple interacting psychological motivations and social factors. Donation behavior is not purely altruistic but rather a mix of altruism, self-interest, emotion, responsibility, and resource exchange. It relates both to individual characteristics and the social environment. Social psychology has made significant contributions to the understanding of donation intention [45]. Based on social psychology theories, this study evaluates the impacts of individual traits, social support, cultural factors, and psychological mechanisms on public donation intention. An analytical framework comprising the empathy–altruism effect, the universal exchange effect, the feedback effect, and the expectancy effect is proposed. This framework aims to elucidate the formation of donation intentions by the public under diverse social and psychological conditions. Within this analytical framework, the hardship level caused by the pandemic can be used to test the empathy–altruism effect, the degree of risk perception can be used to test the universal exchange effect, community material support can be utilized to test the feedback effect, and the evaluation of the pandemic response can be used to test the expectancy effect. The Section 3.2 provides the conceptualization and theoretical derivation of the empathy–altruism effect, universal exchange effect, feedback effect, and expectancy effect within this framework. Refer to Figure 1 for the analytical framework.

### 3.2. Research Hypotheses

Theoretical studies and practical experiences indicate that individual donation intention or behavior is positively correlated with individual income or household economic level [46], representing a rational donation behavior of “acting according to one’s means” under normal circumstances. However, during the COVID-19 pandemic, many people demonstrated great generosity and compassion through donations and volunteer work [47], even facing personal hardships. What drives the high enthusiasm for public donations following the pandemic outbreak? Psychologists believe that altruistic behaviors, such as donations, are based on empathy for the unfortunate situations of others, proposing the empathy–altruism hypothesis. Emotional responses, like sympathy towards others in misfortune, can inspire altruistic behaviors [48]. Emotions enhance empathy [49], allowing individuals to more profoundly empathize with others facing similar difficulties. This deeper empathy, in turn, motivates contributions aimed at mitigating such adversities [50]. In the context of a major public health emergency like the COVID-19 pandemic, individuals empathize with the difficulties and challenges others face. Those whose lives are significantly affected by the pandemic can deeply understand the hardships caused, and their stronger empathy leads to a greater donation intention to help others and overcome difficulties together. Drawing from the empathy–altruism effect, Hypothesis 1 is formulated:

**Hypothesis** **1:** *Members of the public with a higher level of hardship caused by the pandemic have a higher donation intention.*

The COVID-19 pandemic is the most rapidly spreading, widest-reaching, and hardest to control public health emergency that has occurred in China since its founding. Unlike disasters with a clear geographical impact, such as floods or earthquakes, the COVID-19 pandemic poses a threat to everyone until effectively controlled, making nationwide anti-pandemic efforts an intrinsic requirement. Risk perception theory suggests that the degree of cognitive awareness of potential threats influences behavioral responses [51]. An increased public perception of the severity and personal threat of the pandemic may enhance their preventive motivation [52], prompting actions to reduce perceived risks. Donations are actions that reduce individual and social risks by supporting pandemic control and relief efforts; existing research has found that heightened perceptions of pandemic threat significantly encourage public donations [53]. When the threat of the pandemic becomes more severe, people under threat tend to pay more attention to and feel empathy for others experiencing the same threat, thus increasing their donation intention [18]. When faced with widespread uncertainty and threats, donations can be seen as a proactive coping strategy. The public, based on their perception of environmental risks, engages in altruistic actions such as donations with the hope of creating a universal network of mutual aid and support in society, thereby reducing both their own and others’ risks. Therefore, an increased perception of risk not only heightens individuals’ concern and precaution against the effects of the pandemic but may also inspire the intention to combat the pandemic through donations. Drawing from the universal exchange effect, Hypothesis 2 is formulated:

**Hypothesis** **2:** *Members of the public with a higher degree of risk perception have a higher donation intention.*

In the early stages of the pandemic outbreak, due to limited production capacity and reserves, medical supplies related to pandemic prevention, such as masks and disinfectants, were in short supply nationwide, with some areas also experiencing shortages of vegetables, meat, and other life essentials. During a crisis, the demand for goods and donations affects individual charitable donation behaviors [54]. In the context of material shortages and pandemic prevention measures, individuals faced significant challenges in accessing materials for pandemic prevention and daily life due to limitations in information, channels, and capabilities. This situation posed serious threats to their health and well-being. Neighborhood committees (village committees) played a crucial role in pandemic control, including information dissemination, personnel management, and material distribution. Initially, some committees overcame difficulties, providing residents with supplies for living and pandemic prevention or linking them to relevant resources. The materials provided by communities mostly came from donations by the government, other organizations, or individuals. Reciprocity theory suggests that the act of giving and receiving in social interactions is based on mutual benefits and gratitude [55]. Upon receiving help and support from society or their community, residents felt a moral or emotional indebtedness, which in turn made them more willing to give back to society or the community through actions such as donations. Drawing from the feedback effect, Hypothesis 3 is formulated:

**Hypothesis** **3:** *Compared to members of the public who have not received community material support, those who have have a higher donation intention.*

When faced with emergency events, governments typically act swiftly to provide funding and emergency services to affected communities. However, in a risk society, relying solely on the political system is inadequate when confronting major public health emergencies. Effective risk management in these situations requires rational cooperation from the public [56]. In the early stages of the COVID-19 outbreak, the public views on government pandemic control measures significantly influenced their support [57]. Public evaluation of government pandemic response reflects their trust in the government, and those with higher trust in the government tend to respond positively to government donation mobilizations [58]. A lack of trust in the government, charitable organizations, and others has a significant negative effect on public donation behavior. According to expectancy theory, the motivation behind individual behavior is determined by the expectation of the outcome of the behavior [59]. In the midst of a major public health crisis, the public believes that donations can directly or indirectly promote better pandemic control by the government, thereby reducing individual and societal risks and losses. Drawing from the expectancy effect, Hypothesis 4 is formulated:

**Hypothesis** **4:** *Members of the public with a higher evaluation of pandemic control have a higher donation intention.*

Giddens’ theory of structuration suggests that individuals are embedded in social structures, with their actions influenced by various societal factors [60]. Combining perspectives of the empathy–altruism effect, the universal exchange effect, the feedback effect, and the expectancy effect, when government pandemic responses are effective and communities provide timely and effective material support, public trust in the government and society may increase, likely fostering feelings of gratitude and acts of reciprocation. For those significantly affected by the pandemic, on the one hand, they can empathize more with others in similar situations; on the other hand, government pandemic measures and community material support have a more significant effect on them. In this context, even if individuals or families are in hardship due to the pandemic, they are more likely to have a higher intention to donate, based on trust in the government and gratitude, as well as empathy for others, hoping to help others and support pandemic control. For members of the public with strong risk perceptions, the government pandemic response and community material support might bring them greater security, trust, and gratitude, enhancing their expectations of reciprocity through universal exchange achieved by donation. Concurrently, positive emotions are contagious [61], and the heightened donation intention among some members of the public can also boost the donation intention of others. Therefore, it is hypothesized that the evaluation of pandemic control moderates the relationship between the hardship level caused by the pandemic and donation intention, as well as between the degree of risk perception and donation intention, with community material support acting similarly. Thus, the following hypotheses are proposed:

**Hypothesis** **5a:** *For members of the public with a higher evaluation of pandemic control, the hardship level caused by the pandemic has a greater positive impact on their donation intention;*

**Hypothesis** **5b:** *For members of the public with a higher evaluation of pandemic control, the degree of risk perception has a greater positive impact on their donation intention;*

**Hypothesis** **6a:** *Compared to members of the public who have not received community material support, those who have experience a greater positive impact of the hardship level caused by the pandemic on their donation intention;*

**Hypothesis** **6b:** *Compared to members of the public who have not received community material support, those who have experience a greater positive impact of the degree of risk perception on their donation intention.*

## 4. Data and Methods

### 4.1. Data Source

**Figure 2:**
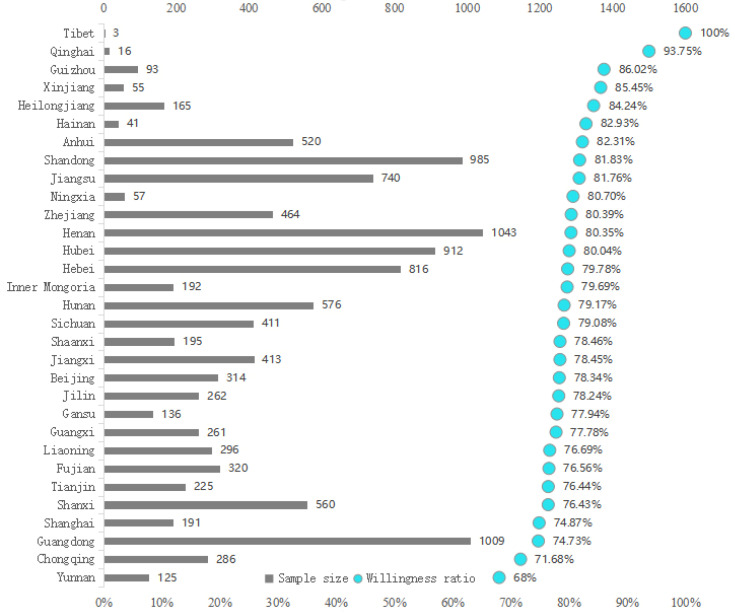
Distribution of data sources.

The data for this study were derived from the questionnaire survey of the research team on “Public Psychology and Behavior during the COVID-19 Pandemic” conducted through “Ruiyan Cloud Survey” from February 9 to February 15 in 2020. The online survey collected 11,000 samples in total. To compensate for potential biases in sample representativeness caused by the online survey, the research team also conducted supplementary surveys via telephone interviews for individuals in remote areas, older age groups, those with lower educational levels, or those who might not have internet access, recovering 745 samples through phone interviews. In total, the research team collected 11,745 samples from urban and rural residents across China. During the data-cleaning process, 63 samples from the online survey were excluded. The exclusion criteria included (1) incomplete questionnaire responses and (2) questionnaire completion times less than 2 min. After data cleaning, the final number of valid samples was 11,682. The samples covered all 31 provinces (municipalities and autonomous regions) of China, targeting Chinese citizens aged 14 and above. Among all respondents, 9225 expressed donation intention, accounting for 78.97% of the total sample, while 3715 engaged in actual donation behavior, representing 31.80% of the total sample. Of those who expressed donation intention, 3223 (34.94%) actually made donations. The source and distribution of the data are shown in Figure 2.

### 4.2. Variable Measurement

#### 4.2.1. Dependent Variable

The dependent variable in this study is donation intention, measured by the item “I am willing to donate money or goods to fight the pandemic” within the question “Please use a 1–5 point scale to evaluate the extent to which each item matches your actual social participation”. Values were assigned from 1 to 5 ranging from “strongly disagree” to “strongly agree”. The overall mean for this item was 4.10 (SD = 1.02), indicating a generally high intention to donate among respondents with responses varying about one scale point from the mean on average. Respondents who chose “relatively agree” or “strongly agree” were considered “yes” and assigned a value of 1, while all other responses were considered “no” and assigned a value of 0.

#### 4.2.2. Independent Variables

The independent variables of this study include the hardship level caused by the pandemic, degree of risk perception, evaluation of the pandemic response, and community material support. The hardship level caused by the pandemic was measured by the scale “Since the outbreak of the pandemic, has your family encountered the following difficulties? Please use a 1–5 point scale to evaluate how each item matches your (family’s) actual situation”, with values assigned from 1 to 5 ranging from “strongly disagree” to “strongly agree”. The overall mean across all items in this scale was 2.91 (SD = 0.84), indicating a moderate level of hardship experienced by respondents with responses varying slightly less than one scale point from the mean on average. This variable’s KMO value was 0.902, and its Cronbach’s alpha was 0.884, suitable for factor analysis. Three factor variables were extracted using factor analysis, with each factor’s variance contribution divided by the cumulative contribution to determine the weights and then weighted summation. The hardship level caused by the pandemic is a dichotomous variable, assigned a value of 1 (high hardship level) if the weighted sum was greater than the mean, or 0 otherwise (low hardship level). The degree of risk perception was measured by the question “Currently, how terrifying do you find the pandemic?” with values assigned from 1 to 5 ranging from “not terrifying at all” to “very terrifying”. The overall mean for this question was 4.38 (SD = 0.74), indicating a generally high level of perceived risk by respondents with responses varying less than one scale point from the mean on average. Respondents who chose “very terrifying” were assigned a value of 1, indicating a high level of fear, while all other responses were assigned a value of 0, indicating a relatively lower level of fear. The evaluation of the pandemic response was constructed from the respondents’ evaluation of pandemic control measures by six levels of organizations from central to local levels, measured by “Currently, how would you evaluate the overall performance of the following levels of institutions in pandemic control?” with evaluations assigned values from 1 to 5 ranging from “very dissatisfied” to “very satisfied”. The overall mean across all items in this scale was 4.00 (SD = 1.01), indicating a generally high level of satisfaction by respondents with responses varying about one scale point from the mean on average. This variable’s KMO value was 0.884, and its Cronbach’s alpha was 0.954, suitable for factor analysis. A single common factor was extracted, and the evaluation of the pandemic response is a dichotomous variable, assigned a value of 1 (high evaluation level) if the common factor score was greater the mean, or 0 otherwise (low evaluation level). The community material support was measured by the question “Regarding this pandemic, what measures have been taken by your village (community) committee (multiple selections allowed)?” Choosing any or multiple options among “linking up with channels for purchasing daily necessities” “distributing alcohol, masks, and other protective materials” or “purchasing and delivering daily necessities on behalf” was assigned a value of 1, or 0 otherwise.

#### 4.2.3. Control Variables

Previous studies have found that individual characteristics including gender, age, marital status, household registration type, years of education, and family characteristics such as family economic status can affect an individual donation intention. The operationalization of each control variable is as follows: gender is a binary variable, with male assigned a value of 1 and female 0; age is an ordinal variable, with ages 14–17 years assigned a value of 1, 18–29 years a value of 2, 30–39 years a value of 3, 40–49 years a value of 4, 50–59 years a value of 5, and 60 years and above a value of 6; marital status is a binary variable, with married (including divorced and widowed) assigned a value of 1 and unmarried 0; household registration type is a binary variable, with agricultural registration as 0 and non-agricultural registration as 1; years of education is an ordinal variable, with elementary school and below assigned 6, junior high school 9, high school/technical secondary school 12, junior college 14, bachelor’s degree 16, and master’s degree and above 18; family economic status is an ordinal variable, with poor, subsistence, well-off, and wealthy sequentially assigned values from 1 to 4.

#### 4.2.4. Interaction Terms

This study hypothesizes that two organizational-level variables, evaluation of the pandemic response and community material support, have moderating effects on two individual-level variables, the hardship level caused by the pandemic and degree of risk perception. Thus, four interaction terms are constructed: the hardship level caused by the pandemic and the evaluation of the pandemic response, the degree of risk perception and the evaluation of the pandemic response, the hardship level caused by the pandemic and community material support, and the degree of risk perception and community material support.

Table 1 lists all the variables used in this study along with their definitions.

### 4.3. Model Construction

This study employs a multivariate logistics regression model to examine the factors influencing public donation intention. The model construction process is as follows:

Suppose the dependent variable donation intention *Y* follows a binomial distribution, with the total probability of *Y* = 1 as *P*(*Y* = 1) and the total probability of *Y* = 0 as *P*(*Y* = 0) = 1 − *P*(*Y* = 1). Then, the multivariate logistics regression model corresponding to n independent variables *X*_1_, *X*_2_, …, *X*_n_ is

(1) $$P(Y=1)=\frac{\exp(\beta_{0}+\beta_{1}X_{1}+\beta_{2}X_{2}+\ldots +\beta_{n}X_{n}+\varepsilon )}{1+\exp(\beta_{0}+\beta_{1}X_{1}+\beta_{2}X_{2}+\ldots +\beta_{n}X_{n}+\varepsilon )}=\frac{1}{1+\exp[-(\beta_{0}+\beta_{1}X_{1}+\beta_{2}X_{2}+\ldots +\beta_{n}X_{n}+\varepsilon )]}$$

Taking the logarithm of both sides yields

(2) $$Logit[P(Y=1)]=\ln[\frac{P(Y=1)}{1-P(Y=1)}]=\beta_{0}+\beta_{1}X_{1}+\beta_{2}X_{2}+\ldots \beta_{n}X_{n}+\varepsilon$$

In the above formula, *X*_1_, *X*_2_, …, *X*_n_ are the independent variables, including control variables (gender, age group, marital status, household registration type, years of education, and family economic status), independent variables (hardship level caused by the pandemic, degree of risk perception, evaluation of the pandemic response, and community material support), and moderating variables (evaluation of the pandemic response and community material support); *β*_1_, *β*_2_, …, *β*_n_ are the regression coefficients for independent variable *X*_i_, with n being the number of independent variables; β_0_ is the intercept, and ε is the random error term.

We used StataMP 17 for data preprocessing and modeling analysis.

## 5. Results

Nested models were employed for regression analysis, with the results presented in Table 2. Model 1 incorporates only control variables, while Model 2 adds independent variables on top of Model 1. Models 3 and 4 are regression models with moderating effects, where different interaction terms are included, respectively, with control variables and independent variables remaining unchanged. Model 3 introduces interaction terms between the hardship level caused by the pandemic and the evaluation of the pandemic response, as well as between the degree of risk perception and the evaluation of the pandemic response, based on Model 2. Model 4 adds interaction terms between the hardship level caused by the pandemic and community material support, and between the degree of risk perception and community material support, building on Model 2.

### 5.1. Baseline Regression Model Analysis

Model 2 indicates that the independent variables—hardship level caused by the pandemic, degree of risk perception, evaluation of the pandemic response, and community material support—all pass the test at a 1% significance level, with positive signs, suggesting a significant positive impact on public donation intention. Hypotheses 1, 2, 3, and 4 are validated. Specifically, with other conditions being equal, members of the public with a higher hardship level caused by the pandemic have a 55.7% higher likelihood of having donation intention compared to those with a lower hardship level. Members of the public with a higher degree of risk perception have a 42.8% higher likelihood of having donation intention compared to those with a lower degree of risk perception. Members of the public with a higher evaluation of the pandemic response have a 103.7% higher likelihood of having donation intention compared to those with a lower evaluation. Members of the public receiving community material support have a 39.2% higher likelihood of having donation intention compared to those without such support.

In terms of control variables, from Model 1 to Model 4, the significance and direction of all control variables remain unchanged, indicating a stable influence of the selected control variables. Regarding individual characteristics, females are more likely to donate than males. Compared to those aged 18 and below, individuals aged 50 and above are less willing to donate. Married individuals are more willing to donate than their unmarried counterparts. The more years spent in public education, the more willing they are to donate. Regarding family economic conditions, the better the public family economic status, the more willing they are to donate. Household registration type does not significantly affect public donation intention, indicating no significant difference in donation intention between rural and urban residents during emergencies.

### 5.2. Regression Model Analysis with Moderating Effects

Table 2, Model 3, shows that the hardship level caused by the pandemic and the degree of risk perception still have a significant positive impact on donation intention. The interaction between the hardship level caused by the pandemic and the evaluation of the pandemic response has a significant positive effect on public donation intention, indicating that the evaluation of the pandemic response enhances the positive impact of the hardship level on public donation intention, thus validating Hypothesis 5a. The interaction between the degree of risk perception and the evaluation of the pandemic response does not significantly affect public donation intention, indicating no significant moderating effect of the evaluation of the pandemic response on the impact of the degree of risk perception on public donation intention, leaving Hypothesis 5b unvalidated. In Model 4, the hardship level caused by the pandemic and the degree of risk perception continue to have a significant positive impact on donation intention. The interaction between the hardship level caused by the pandemic and community material support significantly positively affects public donation intention, indicating that community material support strengthens the positive impact of the hardship level on public donation intention, validating Hypothesis 6a. The interaction between the degree of risk perception and community material support does not significantly affect public donation intention, indicating no significant moderating effect of community material support on the impact of the degree of risk perception on public donation intention, leaving Hypothesis 6b unvalidated. Overall, the evaluation of the pandemic response and community material support significantly and positively moderate the relationship between the hardship level caused by the pandemic on public donation intention. However, they do not have a similar moderating effect on the relationship between the degree of risk perception and public donation intention.

**Figure 3:**
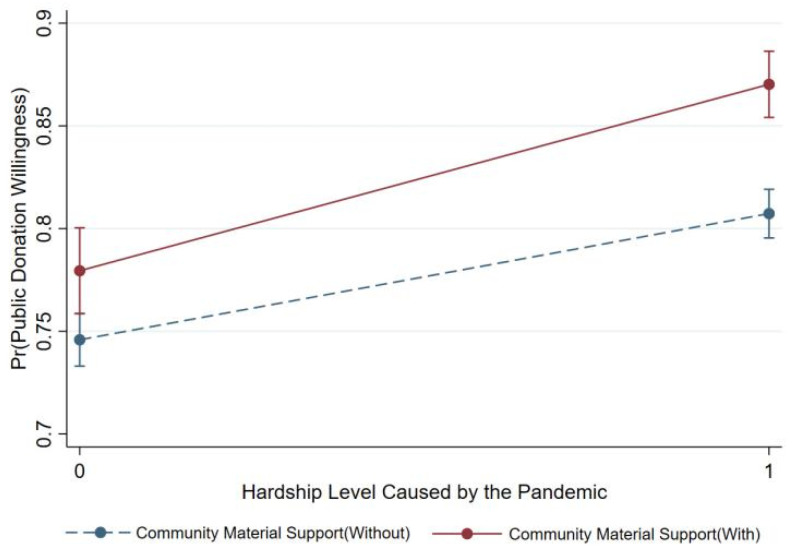
The moderating effect of evaluation of the pandemic response.

**Figure 4:**
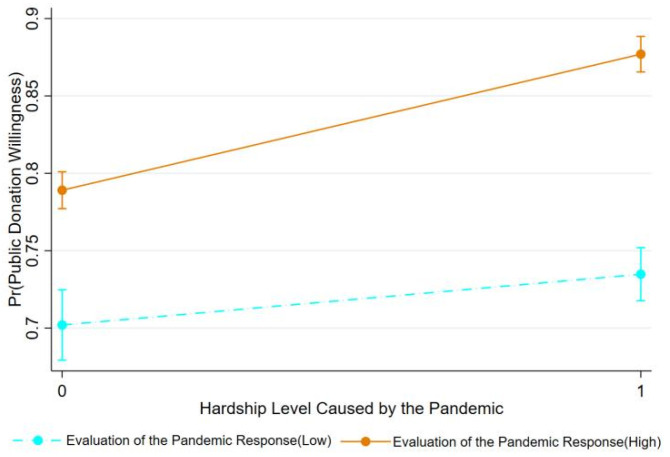
The moderating effect of community material support.

Following Brambor’s analysis method for interaction models [62], moderating effect diagrams for the evaluation of the pandemic response on the hardship level caused by the pandemic and for community material support on the hardship level were drawn. Figure 3 shows that among members of the public with a high evaluation of the pandemic response, the regression slope and intercept for the hardship level caused by the pandemic on public donation intention are greater than for those with a low evaluation, indicating a stronger effect of the hardship level on public donation intention under a high evaluation of the pandemic response. The moderating role of community material support follows the same principle as shown in Figure 4.

## 6. Conclusions and Discussion

### 6.1. Conclusions

During major public health emergencies like the COVID-19 pandemic, widespread public donations can offer substantial help in crisis management on a global scale. This study proposes an analytical framework and examines the factors influencing public donation intention during major public health emergencies and their interactions, composed of the empathy–altruism effect, the universal exchange effect, the feedback effect and the expectancy effect. Based on the questionnaire data from the “Public Psychology and Behavior Study During the COVID-19 pandemic”, multiple logistic regression and moderation effect models were used to analyze these influencing factors and their interactions on public donation intention. This study reveals that among the surveyed sample, 79% of respondents expressed donation intention, indicating a high level of public engagement during the pandemic. Further analysis shows that the hardship level caused by the pandemic, degree of risk perception, community material support, and evaluation of the pandemic response all have significant positive impacts on public donation intention. These findings underscore the importance of transparent communication about the crisis severity, effective risk communication strategies, robust community support systems, and efficient pandemic management in encouraging public donations during global health emergencies. Specifically, (1) the higher the hardship level caused by the pandemic, the more willing the public is to donate. The pandemic leads to difficulties in the lives of the public, and the more severe these difficulties are, the more acutely the public can empathize with the impacts of the pandemic, thus showing a higher donation intention. (2) The higher the degree of risk perception, the more willing the public is to donate. An increase in risk perception helps heighten individual attention to and precaution against the impacts of the pandemic, thereby enhancing the donation intention to fight the pandemic. (3) Material support provided by communities helps increase the public donation intention. During the pandemic, it is more challenging for the public to access materials for daily life and pandemic prevention. Those who receive community material support are more willing to donate out of gratitude and the desire to give back. (4) The higher the evaluation of the pandemic response, the higher the public donation intention. Better government measures and outcomes in controlling the pandemic contribute to gaining public trust, thereby strengthening the public donation intention to support government pandemic prevention mobilizations. (5) The evaluation of the pandemic response and community material support both positively moderate the relationship between the hardship level caused by the pandemic and donation intention, as well as between the degree of risk perception and donation intention. Both the evaluation of the pandemic response and community material support reflect the performance of public institutions in controlling the pandemic. Better performance can enhance public empathy, trust, and gratitude, thereby increasing the donation intention.

The global nature of the COVID-19 pandemic has demonstrated the interconnectedness of our world and the need for international solidarity. This study not only sheds light on the factors influencing donation intention in China during the COVID-19 pandemic but also offers valuable lessons for fostering global community engagement in future public health emergencies. As we face increasingly complex global challenges, understanding and promoting public donation intention will be crucial in building resilient and supportive communities worldwide. By understanding the mechanisms that drive public donations during global crises, governments and organizations worldwide can develop more effective strategies to mobilize resources and foster a spirit of public solidarity in times of crisis.

### 6.2. Discussion

This study provides empirical support for the management of major public health emergencies, highlighting the importance of social–psychological factors in promoting public participation and support for pandemic control efforts. In today’s risk society, when facing similar public health emergencies in the future, it is crucial for governments and communities to enhance the scientific basis, precision, and effectiveness of pandemic prevention policies. Additionally, strengthening both the material and psychological support provided to residents is essential. Therefore, encouraging public participation and leveraging the power of the public to jointly face the crisis are crucial.

While this study provides valuable insights into public donation intentions during public health emergencies in China, it is crucial to consider how these findings might apply to other cultural contexts. Cross-cultural research indicates significant variations in donation behaviors across societies, influenced by cultural values, social norms, and economic factors [47]. Overall, economic and political factors play a more important role than cultural factors in explaining cross-national differences in donation [63]. Therefore, the evaluation of pandemic response may have different impacts on countries with different political systems. The hardship level caused by the pandemic likely has a more universal effect on donation intention across cultures, as the experience of hardship often enhances empathy and prosocial behavior [64]. The impact of risk perception on donation intention may vary considerably across cultures, with cultural values and societal norms playing a crucial role in shaping risk perception and subsequent behaviors [65]. In individualistic cultures like the United States, while risk perception tends to be more personally oriented, the strong emphasis on personal responsibility often promotes prosocial behaviors. This sense of individual accountability can lead to increased charitable donations and volunteerism [66]. In European cultures, characterized by diverse national identities and a strong welfare state tradition, many expect greater state intervention and less reliance on private donations during crises [64]. Nevertheless, the European emphasis on social solidarity might align with the community support factors identified in our study. These cross-cultural considerations underscore the need for cautious interpretation and application of our findings in diverse global contexts.

Despite offering deep insights into the public donation intention during major public health emergencies, this study has its limitations. First, this study cannot establish strict causal relationships. Due to the use of cross-sectional data collected through online questionnaires and telephone interviews, we are unable to conduct experimental analysis or employ complex economic models, which limits our capacity to infer causal relationships between variables. Second, Hypotheses 5b and 6b were not supported by empirical testing. This situation could be due to various factors, including theoretical oversimplifications, methodological limitations, and other influencing factors not considered, which require more in-depth analysis in future research. Third, due to the objective constraints during the initial outbreak of the pandemic, all data mainly came from online questionnaires, and although some telephone surveys were conducted as a supplement, it is still difficult to compensate for the representational error of the sample. Finally, this study does not reveal the gap between donation intention and actual donation behavior. While this study primarily focused on donation intention, we recognize that intention does not always directly translate into action. The transformation of intentions into behavior is influenced by several factors, including resource endowments [6], goal dimensions, the basis of the intention, and properties of the intention [8]. Future research and policy design can focus on these aspects and use more diversified data and methods to improve the understanding of the mechanisms affecting public donation intention and behavior during extraordinary times. This could include exploring the dynamics of the intention–behavior relationship, identifying factors that facilitate or hinder the actualization of donation intentions, and investigating the long-term impacts of crisis experiences on donation patterns.

## Figures and Tables

**Table 1:** Variable definitions and assignments.

Variable Type	Variable Name	Assignment/Value Range	Percentage (%)
Dependent Variable	Donation Intention	No = 0	21.03
		Yes = 1	78.97
Control Variables	Gender	Female = 0	44.49
		Male = 1	55.51
	Age Group	14–17 years = 1	3.96
		18–29 years = 2	60.72
		30–39 years = 3	21.00
		40–49 years = 4	7.34
		50–59 years = 5	3.33
		60 years and above = 6	3.65
	Marital Status	Unmarried = 0	60.53
		Married = 1	39.47
	Household Registration Type	Agricultural registration = 0	52.81
		Non-Agricultural registration = 1	47.19
	Years of Education	Elementary school and below = 6	3.39
		Junior high school = 9	6.88
		High school/Technical secondary school = 12	16.54
		Junior college = 14	21.30
		Bachelor’s degree = 16	42.00
		Master’s degree and above = 18	9.89
	Family Economic Status	Poor = 1	12.12
		Subsistence = 2	56.40
		Comfortable = 3	30.05
		Wealthy = 4	1.42
Independent Variables	Hardship Level Caused by the Pandemic	Low = 0	51.57
		High = 1	48.43
	Degree of Risk Perception	Low = 0	50.57
		High = 1	49.43
	Community Material Support	Without = 0	72.42
		With = 1	27.58
	Evaluation of the Pandemic Response	Low = 0	34.64
		High = 1	65.36

**Table 2:** Estimation results of logistic regression models on factors influencing public donation intention during major public health emergency (N = 11,682).

Variables	Model 1	Model 2	Model 3	Model 4
	Odds Ratio	Odds Ratio	Odds Ratio	Odds Ratio
Control Variables				
Gender (Female)	0.736 ***	0.750 ***	0.753 ***	0.750 ***
Age Group (14–17 years)				
18–29 years	0.974	0.922	0.934	0.923
30–39 years	0.916	0.867	0.877	0.869
40–49 years	0.861	0.843	0.843	0.844
50–59 years	0.592 ***	0.602 ***	0.605 ***	0.603 ***
60 years and above	0.564 ***	0.630 ***	0.641 **	0.627 ***
Marital Status (Unmarried)	1.321 ***	1.219 **	1.220 **	1.218 **
Household Registration Type (Agricultural Registration)	0.972	1.036	1.035	1.033
Years of Education	1.089 ***	1.104 ***	1.102 ***	1.105 ***
Family Economic Status	1.169 ***	1.209 ***	1.209 ***	1.209 ***
Independent Variables				
Hardship Level Caused by the Pandemic (Low)		1.557 ***	1.184 **	1.455 ***
Degree of Risk Perception (Low)		1.428 ***	1.479 ***	1.423 ***
Community Material Support (Without)		1.392 ***	1.396 ***	1.211 **
Evaluation of the Pandemic Response (Low)		2.037 ***	1.665 ***	2.040 ***
Interaction Terms				
Hardship Level Caused by the Pandemic × Evaluation of the Pandemic Response			1.642 ***	
Degree of Risk Perception × Evaluation of the Pandemic Response			0.926	
Hardship Level Caused by the Pandemic × Community Material Support				1.343 ***
Degree of Risk Perception × Community Material Support				1.02
Constant	0.959	0.307 ***	0.361 ***	0.315 **
Pseudo R2	0.021	0.052	0.054	0.052

Note: *** and ** denote significance at the 1% and 5% levels, respectively.

## Data Availability

The data presented in this study are available on request from the corresponding author due to privacy and ethical restrictions. The dataset contains sensitive information about living conditions, behaviors, attitudes, and evaluations related to the COVID-19 pandemic from 11,682 participants in China. To protect the privacy of the participants and prevent potential misuse of the data, public access to the dataset is restricted. Access to the data will be granted to qualified researchers who provide a detailed research proposal and agree to the terms of a data sharing agreement that ensures the proper use of the data.
